# Losing weight, gaining confidence? actual weight does not predict body (dis)satisfaction and self-esteem in adolescents with anorexia nervosa

**DOI:** 10.1186/s40337-025-01338-3

**Published:** 2025-07-16

**Authors:** Linda Lukas, Laura Nuding, Gerd Schulte-Körne, Belinda Platt, Anca Sfärlea

**Affiliations:** Department of Child and Adolescent Psychiatry, Psychosomatics and Psychotherapy, University Hospital, LMU Munich, Nussbaumstr. 5, 80336 Munich, Germany

**Keywords:** Eating disorders, Adolescence, Cognitive biases, Negative biases, Self-esteem, Weight, Body dissatisfaction

## Abstract

**Background:**

In line with modern-day beauty ideals, many adolescent girls strive to have a slender figure. Thus, low body weight is expected to be related to greater body satisfaction and self-esteem in adolescent girls. However, for adolescents with anorexia nervosa (AN) this is often not the case: despite being underweight, they exhibit high levels of body *dis*satisfaction and low self-esteem. Negative cognitive biases for information related to one’s body might explain this disconnection between body weight and body (dis)satisfaction/self-esteem in adolescents with AN. The present study explores the association between actual weight, negative cognitive biases, and body dissatisfaction/self-esteem in both adolescents with AN and adolescents without mental disorder (healthy controls; HCs).

**Methods:**

Weight was assessed as Body Mass Index Standard Deviation Score, interpretation bias for body-related information was assessed with an experimental paradigm (Scrambled Sentences Task), body dissatisfaction was measured using the Body Shape Questionnaire, and self-esteem was measured using the Rosenberg Self-Esteem Scale in *n* = 40 12-18-year-old adolescent girls with AN and *n* = 40 HCs. Hierarchical regression analyses were calculated to investigate whether weight and/or interpretation biases predicted body dissatisfaction and self-esteem.

**Results:**

In adolescents with AN, negative body-related interpretation bias was a significant positive predictor of body dissatisfaction and negative predictor of self-esteem while body weight did not predict any of the outcome measures. In HCs, both weight and negative interpretation bias were significant positive predictors of body dissatisfaction and significant negative predictors of self-esteem.

**Conclusion:**

The results show a disconnection between body weight and body (dis)satisfaction/self-esteem in adolescents with AN and highlight the association between negative cognitive biases for body-related information and body (dis)satisfaction/self-esteem. The negatively biased processing of information related to one’s body could confirm the perception of the body as flawed and not thin enough and hinder adolescents with AN to perceive their bodies’ thinness, even in the state of severe underweight. The results underline the need to target negatively biased cognitions about the body in AN treatment.

**Supplementary Information:**

The online version contains supplementary material available at 10.1186/s40337-025-01338-3.

## Abstract (plain language summary)

In line with societal beauty ideals, many adolescent girls strive to have lean bodies and thus lower body weight is expected to be related to higher body satisfaction and self-esteem. However, this does not apply to all adolescent girls, especially not to those with anorexia nervosa, an eating disorder characterized by persistent drive for thinness and body dissatisfaction despite being underweight or having lost a significant amount of weight. This might be explained by negatively biased thoughts about their bodies that affect how individuals with anorexia nervosa perceive and evaluate themselves. The present study investigates how actual body weight and negatively biased thoughts about the body relate to body (dis)satisfaction and self-esteem in adolescent girls with anorexia nervosa and girls without mental disorder. Among girls without a mental disorder, both lower body weight and less negative thoughts about their bodies were associated with greater body satisfaction and self-esteem. In contrast, among girls with anorexia nervosa, body satisfaction and self-esteem were associated with less negative thoughts about their bodies but *not* with their actual weight. This underlines the relevance of negative thinking patterns in adolescents with anorexia nervosa as well as the need to address these patterns in treatment and prevention.

**Supplementary Information:**

The online version contains supplementary material available at 10.1186/s40337-025-01338-3.

## Background

Despite body positivity movements gaining popularity, the expectation to conform to ideals of beauty remains strong in today’s image-driven society [1], where social media platforms are flooded with filtered and edited images that propagate and perpetuate unrealistic ideals [2–4]. Adolescents are confronted daily with idealized representations of beauty and thinness [2, 3, 5], fostering altered self-perception [6], internalization of beauty ideals [7], and appearance comparison to unrealistic pictures of others [7, 8]. This in turn leads to increased body dissatisfaction and self-esteem concerns, especially among females [9–16]. Many adolescent girls do not meet these unrealistic ideals but even those who do have a thin body are not necessarily satisfied with it. It seems that for some girls, being thin fails to boost self-esteem and body satisfaction – despite achieving the societal ideal. This applies particularly to individuals with anorexia nervosa (AN), a psychiatric condition characterized by significantly low body weight achieved by food restriction and/or excessive exercise, an intense fear of gaining weight, and a distorted body image [17], resulting in many individuals with AN still feeling “fat” even in a state of severe emaciation [18, 19]. In many individuals with AN this distorted body image is accompanied by a general lack of insight regarding the severity of their condition [20–22]. The present study investigates how, for adolescents with AN, even severe emaciation is not experienced as ‘thin enough’ [17], i.e., why the drive for thinness and body dissatisfaction persist despite substantial weight loss.

The incidence of AN among adolescents has increased in recent decades [23, 24], with a particularly sharp rise in hospital admissions during and after the COVID-19 pandemic [25–29]. An early onset of the disorder is associated with poorer outcomes [30], higher symptom severity [31], increased life difficulties [32], and greater psychiatric comorbidity throughout life [31]. Understanding the mechanisms driving the development and maintenance of AN during this critical developmental period is crucial for effective prevention, early intervention, and treatment strategies.

Low self-esteem and high body dissatisfaction are pivotal risk factors for eating disorders [33–35] that are closely linked [36, 37] and mutually reinforce each other in both non-clinical samples [37, 38] and individuals with eating disorders [39]. Self-esteem refers to an individual’s overall sense of self-worth or personal value [40]. Body dissatisfaction refers to the negative feelings that individuals have about their body size, shape, or weight [41] and is driven by a discrepancy between their desired versus actual body [42]. These risk factors might play a particularly important role in adolescents, who are in a critical stage of identity formation [43] and subject to rapidly changing perceptions, thoughts, and feelings about their developing bodies [44]. This leaves them especially vulnerable to constant comparison to the unrealistic images portrayed in the media, societal standards that equate thinness with beauty and success [45, 46], and pressures to correspond to a certain body ideal [47]. Adolescents who do not meet this ideal often experience not only high levels of body dissatisfaction (which affect approximately 26–61% of female youth [48]) but also a decline in self-esteem, which for many adolescents is closely tied to their body weight and shape, a phenomenon known as the “body weight contingency of self-worth” [49]. Both can lead to restrictive eating and other, potentially unhealthy, weight control behaviors such as extreme dieting, excessive exercise, or even purging [34, 50, 51]. These behaviors are intended to reduce weight in order to achieve the desired body and thereby enhance body satisfaction and self-esteem [52–54], but can be harmful and put adolescents at risk for the development of eating disorders [55–57].

Body weight is not always a reliable predictor of body satisfaction and self-esteem: whereas for girls without mental disorder achieving a lower body weight is associated with higher self-esteem and body satisfaction [37, 58–60], body weight seems to be decoupled from body satisfaction and self-esteem in adolescents with AN [39, 61]. Despite reaching or even exceeding ideals of thinness, individuals with AN often continue to experience low self-esteem and high body dissatisfaction [18, 19, 62–64]. This paradox raises the question as to what other psychological or cognitive factors predict self-esteem and body (dis)satisfaction in adolescents with AN.

Cognitive biases might play an important role in perpetuating the cycle of low self-esteem and body dissatisfaction [65, 66] in individuals with AN. Cognitive biases are tendencies to preferentially process information that is congruent with one’s maladaptive cognitive schemata [66], which in the case of AN often include increased concern with body weight and shape as well as excessive body weight contingency of self-worth [65, 67]. In contrast to body (dis)satisfaction that is assessed via questionnaires and represents an explicit and conscious evaluation of one’s body, cognitive biases are thought to occur automatically and are assessed via more implicit measures [68]. Cognitive biases are found at different levels of information processing in both adults and adolescents with AN, particularly when they are confronted with information related to their bodies. Adolescents with AN (compared to those without mental disorder) show increased attention towards “unattractive” body parts when looking at pictures of their bodies (i.e., show negative attention biases [69–71]), interpret ambiguous information related to their bodies negatively (i.e., show negative interpretation biases [72, 73]), and remember negative information related to their bodies better than positive information (i.e., show negative memory biases [73, 74]). This biased information processing confirms the perception of one’s body as flawed and not thin and attractive enough, thereby reinforcing the maladaptive cognitive schemata and contributing to the maintenance and aggravation of eating disorder symptoms. Thus, cognitive biases might explain why high body dissatisfaction and low self-esteem are persistent and even increased in adolescents with AN even though they have reached the ideal of thinness.

In our previous study [73], we found adolescents with AN to show more negative interpretation biases for information related to one’s body as well as higher body dissatisfaction and lower self-esteem compared to a healthy control (HC) group of adolescents. Interpretation biases, body dissatisfaction, and self-esteem were highly correlated. The present additional analysis explores to what extent actual body weight vs. interpretation biases explain body dissatisfaction and self-esteem in adolescents with AN, compared to HCs. To the best of our knowledge, no study so far has addressed this question. However, the aforementioned considerations and empirical results give rise to the assumption that actual body weight and interpretation biases play different roles in adolescents with AN vs. HCs. In HCs, we expect actual body weight to be a positive predictor for body dissatisfaction and a negative predictor for self-esteem. In adolescents with AN, we do not expect weight to be a predictor or body satisfaction or self-esteem. Instead, we expect interpretation bias for body-related information to be a positive predictor for body dissatisfaction and a negative predictor for self-esteem.

## Methods

The presented data were collected as part of a study on cognitive biases in adolescents with AN (KOALA study)^1^ [73, 75].

### Participants

Forty adolescent girls with AN and 40 HC girls without mental disorder aged 12–18 years were included in the present study. Adolescents with AN were recruited in the Department of Child and Adolescent Psychiatry, Psychosomatics and Psychotherapy at the LMU University Hospital Munich. HC girls were recruited by contacting girls who had taken part in previous studies in the Department as controls and inviting them to participate in this study.

For all participants, the presence of current and past psychiatric diagnoses were assessed using a standardized, semi-structured clinical interview (Kinder-DIPS [76, 77]). The Kinder-DIPS is a well-validated [76, 78] diagnostic interview that allows diagnosis of a wide range of psychiatric disorders according to DSM-5 [17] in children and adolescents. The interview was conducted with the participants by trained interviewers. Interrater-reliability in our sample was good, with accordance rates ≥ 93% for lifetime diagnoses of AN, major depression, social phobia, and generalized anxiety disorder [73]. Participants were included in the AN group if they met criteria for AN (including atypical AN) according to DSM-5 [17]; participants were included in the HC group if they did not meet criteria for any current or past mental disorder according to the Kinder-DIPS. Exclusion criteria for all groups were: below average intelligence (IQ < 85, measured with the first part of the Culture Fair Intelligence Test (CFT-20-R), a language-free test measuring general fluid intelligence [79])^2^, insufficient German language skills, non-corrected visual impairment, pervasive developmental disorders, psychotic or bipolar disorders, or substance abuse.

Sample characteristics are presented in Table 1. Most adolescents with AN had AN of the restricting type and were in the acute state of the disorder, i.e., underweight (BMI < 10th age-corrected BMI-percentile), while *n* = 3 had atypical AN or were partially weight-remitted (BMI > 10. age-corrected BMI-percentile). The groups differed in weight (i.e., BMI and -percentile/-SDS), eating disorder psychopathology (assessed with the Eating Disorder Inventory-2, EDI-II [80, 81]), body dissatisfaction, self-esteem, as well as cognitive biases. For further details about illness duration, comorbidities, or medication of the AN sample see Lukas et al. [73].

**Table 1:** Demographic and clinical characteristics

	AN *n* = 40				HC *n* = 40				
	M	(SD)	Range		M	(SD)	Range	t	*p*
Age	15.50	(1.55)	12.33–18.67		15.74	(1.80)	12.08–18.50	0.65	0.111
BMI	16.14	(1.37)	13.20–20.70		21.03	(2.95)	15.60–31.50	9.51	< 0.001
Age-corrected BMI-Percentile	5.00	(8.56)	0–52		54.00	(24.09)	3–99	12.11	< 0.001
BMI-SDS	-2.02	(0.79)	-5.01–0.04		0.17	(0.81)	-1.83–2.60	12.22	< 0.001
Eating disorder symptoms (EDI)	303.43	(73.00)	141–455		179.68	(40.15)	121–287	9.07	< 0.001
Body dissatisfaction (BSQ)^a^	133.72	(41.44)	49–194		57.13	(27.73)	34–164	9.68	< 0.001
Self-esteem (RSES)^b^	12.67	(7.37)	1–29		25.31	(5.49)	6–30	8.63	< 0.001
Interpretation bias^c^	0.60	(0.33)	0.04–1.00		0.128	(0.24)	0.00–0.96	7.24	< 0.001

Note. AN = anorexia nervosa; BMI = Body Mass Index; BMI-SDS = Body Mass Index Standard Deviation Score; BSQ = Body Shape Questionnaire; EDI = Eating Disorder Inventory-2; HC = healthy control; RSES = Rosenberg Self-Esteem Scale

^a^ available from *n* = 39 AN and *n* = 40 HC participants

^b^ available from *n* = 40 AN and *n* = 39 HC participants

^c^ available from *n* = 40 AN and *n* = 39 HC participants

### Measures

Body weight was operationalized as relative to adolescents’ age and height, i.e., as Body Mass Index Standard Deviation Score (BMI-SDS). For participants in the AN group, height and weight were obtained from their medical records measured by a physician in the week of the data collection. Participants in the HC group were measured and weighed in our laboratory. BMI was calculated and age-corrected BMI percentile and BMI-SDS were derived based on normative data [82].

Body dissatisfaction was assessed with the German version of the Body Shape Questionnaire (BSQ [83]) which is a valid and reliable measure of broadly defined body dissatisfaction [82, 84]. It consists of 34 items that can be answered on a 6-point Likert scale ranging from 1 = “never” to 6 = “always”. Scores from all items are summed up to a total score with higher scores representing greater body dissatisfaction. Internal consistency in our sample was excellent (Cronbach’s α = 0.99).

Self-esteem was assessed using the German version of the Rosenberg Self-Esteem Scale (RSES [85]), which is well validated [85, 86]. It consists of 10 items that are rated on a 4-point Likert scale from 0 = “strongly disagree” to 3 = “strongly agree”. Higher total scores represent higher self-esteem. Internal consistency in our sample was excellent (Cronbach’s α = 0.95).

Interpretation biases for information related to one’s body were assessed using the Scrambled Sentences Task (SST; computerized version adapted from [87, 88]). The SST determines interpretation biases as a tendency to form either negative or positive sentences out of ambiguous information and was initially designed to assess depression-related interpretation biases [89] and later adapted for interpretation biases related to other disorders including eating disorders (e.g [72, 90]). It is considered a valid and reliable measure [91, 92]. The SST used in the present study consisted of 70 scrambled sentences out of three categories (body-related emotional sentences, non-body-related emotional sentences, and neutral sentences) of which only the body-related emotional sentences were relevant in the present investigation. This category comprised 28 scrambled sentences that were based on the stimulus set by Brockmeyer et al. [72] and included self-referent sentences related to one’s body, weight, and physical appearance (e.g., “my fat bottom find attractive I”, “body others appealing my repulsive find”). The scrambled sentences were presented on a computer screen and the participants were asked to order 5 of the 6 presented words into a grammatically correct sentence. Each trial allowed one positive (“I find my bottom attractive”, “Others find my body appealing”) and one negative (“I find my bottom fat”, “Others find my body repulsive”) solution. To better capture implicit processes, the task was performed under cognitive load (memorizing 5-digit numbers throughout the task) and with a time limit. Interpretation bias scores were determined as the ratio of negative sentences relative to all correctly built sentences. Validity of the task in our study is reflected by high correlations of the interpretation bias score with eating disorder symptoms and body dissatisfaction. Split-half and interrater reliabilities were determined as correlations of bias scores based on odd vs. even trials or from two independent raters and were excellent (*r*s ≥ 0.94 [73]). The SST was followed by an incidental free recall task in which participants were asked to recall as many of the sentences they had previously formed as possible to additionally assess memory biases. Due to this study design, memory biases were not independent from interpretation biases and interpretation and memory bias scores were highly correlated (*r* =.81 [73]) in the present study. Therefore, results with memory bias as a measure for cognitive biases were not included in the main analyses but are reported in the supplement.

### Data analysis

All analyses were performed using SPSS 28. To examine relations between BMI-SDS, self-esteem, body dissatisfaction, and interpretation bias scores, pearson correlations were calculated separately for the two groups. Hierarchical linear regressions were used to determine to what extent variations in weight and body-related interpretation bias (predictors) explained variations in self-esteem and body dissatisfaction (outcome variables). The first regression analyses were calculated on all participants and included group × BMI-SDS as well as group × interpretation bias interactions. In the first step, BMI-SDS was included as a predictor; in the second step, the interpretation bias score was added; and in the third step the two interaction terms were included as well. To further explore different patterns in the groups, separate hierarchical regression analyses for each group were then conducted. Within the HC group, two participants were identified as statistical outliers in the regression analyses based on the recommendation of interpreting the standardized and studentized residuals [93] and therefore were excluded from all regression analyses. As BMI-SDS was used for the analyses, floor effects were not an issue and normal distribution of the scores was given in both groups, together with all other assumptions for regression analyses. For all effects, the level of significance was set to *p* =.05 (two-tailed).

## Results

### Correlations

Correlations are presented in Table 2. In the AN group, interpretation bias correlated negatively with self-esteem and positively with body dissatisfaction while self-esteem and body dissatisfaction correlated negatively. None of these variables correlated significantly with BMI-SDS. In the HC group, similar correlations between interpretation bias, self-esteem, and body dissatisfaction emerged. In addition, we observed significant positive correlations between BMI-SDS and self-esteem as well as body dissatisfaction.

**Table 2:** Pearson correlations

	Weight (BMI-SDS)	Self-esteem (RSES)	Body dissatisfaction (BSQ)
***AN***			
Weight (BMI-SDS)			
Self-esteem (RSES)	0.06		
Body dissatisfaction (BSQ)	-0.12	-0.73***	
Interpretation bias	-0.24	-.75***	0.70***
***HC***			
Weight (BMI-SDS)			
Self-esteem (RSES)	-0.32**		
Body dissatisfaction (BSQ)	0.46**	-0.52***	
Interpretation bias	-0.00	-0.49**	0.35*

Note. AN = anorexia nervosa; BMI-SDS = Body Mass Index Standard Deviation Score; BSQ = Body Shape Questionnaire; HC = healthy control; RSES = Rosenberg Self-Esteem Scale

*p <.05, **p <.01, ***p <.001

### Hierarchical regression analyses

For body dissatisfaction, the results of the regression analyses including all participants revealed that BMI-SDS alone accounted for a significant amount of variance in body dissatisfaction (*F*_1,74_ = 35.37, *p* <.001, *R²* = 0.31). When interpretation bias was added into the model, the explained variance increased significantly (*ΔR²* = 0.31, *p* <.001; *F*_2,73_ = 63.45, *p* <.001, *R²* = 0.63). When the interaction terms were included, the model again significantly improved (*ΔR²* = 0.10, *p* <.001; *F*_4,71_ = 49.67, *p* <.001, *R²* = 0.72). Crucially, both interaction effects were significant (see Table 3), indicating that both the relationships between BMI-SDS and body dissatisfaction as well interpretation bias and body dissatisfaction differed significantly by group. The same pattern emerged with self-esteem as dependent variable: BMI-SDS alone accounted for a significant amount of variance in self-esteem (*F*_1,74_ = 38.19, *p* <.001, *R²* = 0.33). When adding interpretation bias into the model, the explained variance increased significantly (*ΔR²* = 0.37, *p* <.001; *F*_2,73_ = 88.42, *p* <.001, *R²* = 0.70). When the interaction terms were included, the model again significantly improved (*ΔR²* = 0.07, *p* <.001; *F*_2,71_ = 62.25, *p* <.001, *R²* = 0.77). The group × interpretation bias interaction was significant while the group × BMI-SDS interaction was a trend (see Table 3).

**Table 3:** Results of the regression analyses across both groups

	B	SE for B	95% CI for B	β	t	*p*
*Body dissatisfaction (BSQ)*						
STEP 1						
Weight (BMI-SDS)	-22.28	3.75	[-29.74, 14.81]	-0.57	-5.95	**< 0.001**
STEP 2						
Weight (BMI-SDS)	-6.48	3.42	[-13.29, 0.33]	-0.17	-1.90	0.062
Interpretation bias	96.41	12.22	[72.06, 120.76]	0.69	7.89	**< 0.001**
STEP 3						
Weight (BMI-SDS)	-28.62	10.56	[-49.67, -7.56]	-0.73	-2.71	**0.008**
Interpretation bias	174.05	31.24	[111.75, 236.35]	1.24	5.57	**< 0.001**
Group × BMI-SDS	21.01	7.24	[6.58, 35.44]	0.69	2.90	**0.005**
Group × Interpretation bias	-71.22	22.51	[-116.11, -26.33]	-0.60	-3.16	**0.002**
*Self-esteem (RSES)*						
STEP 1						
Weight (BMI-SDS)	3.90	0.63	[2.64, 5.15]	0.58	6.18	**< 0.001**
STEP 2						
Weight (BMI-SDS)	1.01	0.52	[-0.02, 2.05]	0.15	1.95	0.055
Interpretation bias	-17.95	1.87	[-21.68, -14.22]	-0.74	-9.58	**< 0.001**
STEP 3						
Weight (BMI-SDS)	3.03	1.66	[-0.27, 6.34]	0.45	1.83	0.071
Interpretation bias	-32.11	4.89	[-41.87, -22.35]	-1.33	-6.56	**< 0.001**
Group × BMI-SDS	-2.25	1.15	[-4.54, 0.03]	-0.43	-1.97	0.053
Group × Interpretation bias	12.27	3.54	[5.21, 19.32]	0.60	3.47	**< 0.001**

Note. BMI-SDS = Body Mass Index Standard Deviation Score; BSQ = Body Shape Questionnaire; RSES = Rosenberg Self-Esteem Scale. Both regression analyses were conducted with *n* = 76 participants, as no BSQ-score was available from one participant in the AN group and no RSES score was available from one participant in the HC group

To further explore the significant interaction we conducted separate hierarchical regression analyses for each group. In the AN group, the regression revealed that BMI-SDS alone did not account for a significant amount of variance in body dissatisfaction (*F* < 1), but when entering interpretation bias, the model was significant (*F*_1,36_ = 17.06, *p* <.001, *R²* = 0.46)^3^ with interpretation bias being a significant positive predictor for body dissatisfaction. The same pattern emerged for self-esteem: BMI-SDS alone did not account for a significant amount of variance in self-esteem (*F *< 1). When interpretation bias was added, the model explained a significant proportion of variance (*F*_1,37_ = 24.84, *p* <.001, *R²* = 0.55) and interpretation bias was a significant negative predictor of self-esteem (see Table 4).

In contrast, in the HC group, BMI-SDS alone accounted for a significant amount of variance in body dissatisfaction (*F*_1,35_ = 9.01, *p* =.005, *R*^*2*^ = 0.18). When interpretation bias was added into the model, the explained variance increased significantly (*ΔR*^*2*^ = 0.14, *p* =.011; *F*_1,34_ = 8.89, *p* <.001, *R²* = 0.31). Both BMI-SDS and interpretation bias were significant positive predictors for body dissatisfaction. BMI-SDS also explained a significant amount of variance self-esteem (*F*_1,34_ = 4.74, *p* =.037, *R*^*2*^ = 0.10). Adding interpretation bias increased the proportion of explained variance significantly (*ΔR*^*2*^ = 0.33, *p* <.001; *F*_1,33_ = 13.86, *p* <.001, *R²* = 0.42). Both BMI-SDS and interpretation bias were significant negative predictors of self-esteem in this final model (see Table 4).

**Table 4:** Results of the regression analyses conducted separately in the two groups

	B	SE for B	95% CI for B	β	t	*p*
***AN*** ^***a***^						
*Body dissatisfaction (BSQ)*						
STEP 1						
Weight (BMI-SDS)	-6.17	8.45	[-23.30, 10.95]	-0.12	-0.73	0.470
STEP 2						
Weight (BMI-SDS)	3.23	6.40	[-9.74,16.20]	0.06	0.50	0.617
Interpretation bias	88.12	15.31	[57.07, 119.17]	0.71	5.76	**< 0.001**
*Self-esteem (RSES)*						
STEP 1						
Weight (BMI-SDS)	0.58	1.50	[7.23, 20.45]	0.06	0.382	0.704
STEP 2						
Weight (BMI-SDS)	-1.17	1.03	[-3.26, 0.911]	-0.13	-1.14	0.261
Interpretation bias	-17.24	2.45	[-22.21, 12.27]	-0.78	-7.03	**< 0.001**
***HC*** ^***b***^						
*Body dissatisfaction (BSQ)*						
STEP 1						
Weight (BMI-SDS)	14.04	4.68	[4.54, 23.54]	0.45	3.00	**0.005**
STEP 2						
Weight (BMI-SDS)	14.25	4.31	[5.48, 23.01]	0.46	3.30	**0.002**
Interpretation bias	40.60	15.14	[9.82, 71.37]	0.37	2.68	**0.011**
*Self-esteem (RSES)*						
STEP 1						
Weight (BMI-SDS)	-1.60	0.736	[25.38, 27.89]	-0.35	-2.18	**0.037**
STEP 2						
Weight (BMI-SDS)	-1.65	0.588	[-2.85, -0.46]	-0.36	-2.81	**0.008**
Interpretation bias	-9.19	2.04	[-13.34, -5.04]	-0.58	-4.50	**< 0.001**

Note. AN = anorexia nervosa; BMI-SDS = Body Mass Index Standard Deviation Score; BSQ = Body Shape Questionnaire; HC = healthy control; RSES = Rosenberg Self-Esteem Scale

^a^ The regression analysis on BSQ-scores was conducted with *n* = 39 participants, as no BSQ-score was available from one participant in the AN group

^b^ The regression analysis on RSES-scores was conducted with *n* = 36 participants, as no RSES-score was available from one participant in the HC group (and two participants were identified as outliers and excluded from all regression analyses)

## Discussion

To the best of our knowledge, this is the first study to investigate the extent to which actual weight (BMI-SDS) and body-related interpretation biases cross-sectionally predict self-esteem and body dissatisfaction in adolescents with AN and HC adolescents. Regression analyses in the total sample showed that both BMI-SDS and interpretation bias were significant predictors of body dissatisfaction and self-esteem, but these associations differed by group (indicated by significant interactions). Therefore, subsequent analyses focusing on group-specific patterns were conducted and showed that in adolescents with AN, weight (BMI-SDS) did not predict self-esteem or body dissatisfaction while body-related interpretation bias was a significant predictor of both. In HC adolescents, both weight (BMI-SDS) and body-related interpretation bias were significant predictors of self-esteem and body dissatisfaction. Of note, the same pattern of results emerged regarding memory bias (see supplement), which was expected since interpretation and memory biases were interdependent and highly correlated in the present study.

In adolescents with AN, actual weight (BMI-SDS) was not related to and did not predict body dissatisfaction or self-esteem. However, adding interpretation bias resulted in a significant model with interpretation bias (i.e., a more negative interpretation of information related to one’s body) being a positive predictor for body dissatisfaction (accounting for 46% of variance) and a negative predictor for self-esteem (accounting for 55% of variance). These results are in line with our hypotheses and support the idea that in adolescents with AN, the evaluation of both the body (in terms of body dissatisfaction) and the self (in terms of self-esteem) is not related to actual weight or the objective physical condition of their bodies. Instead, it is strongly related to negatively biased cognitions about their bodies. These cognitive biases may distort how adolescents with AN perceive their bodies, preventing them from experiencing their bodies as thin, even when they are underweight, and may reinforce their belief that their bodies are flawed, unattractive, and not thin enough, thereby perpetuating their high body dissatisfaction and low self-esteem. This finding aligns with prior research showing that individuals with AN are characterized by negative self-perceptions and body dissatisfaction despite being underweight [18, 19, 62–64]. Of note, malnutrition, which characterizes adolescents with AN in the acute state of the disorder, is associated with increased rigidity and impaired cognitive flexibility [95], which may contribute to the cognitive biases observed in this population. Therefore, cognitive biases (like cognitive flexibility) might improve with renourishment. Other studies have found dysfunctional cognitions to be as pronounced in individuals with atypical AN as in individuals with typical AN, despite the former group having a healthier weight [96]. As our sample consisted mainly of individuals with AN of the restricting type in the acute phase of the disorder, we do not know if the results generalize to atypical or weight-restored AN samples.

Our findings contrast with the results of Brockmeyer et al. [52], who found that in adults with AN, lower body weight – mediated through achievement satisfaction – was associated with higher self-esteem during the acute phase of the disorder. Thus, it seems that weight loss might momentarily bolster self-esteem in adults with AN, whereas in adolescents biased cognitions might overshadow the impact of the actual physical condition of the body. This difference might be explained by (i) increased preoccupation with appearance in adolescence [42, 97]; (ii) brain maturation and hormonal changes associated with an enhanced emotional sensitivity [98], which could leave adolescents particularly susceptible to negative cues in ambiguous emotional information and result in particularly pronounced cognitive biases; (iii) differing illness durations (with presumably longer illness durations in the adult sample of Brockmeyer et al. [52] than in the present adolescent sample); or (iv) methodological differences between the studies. Future longitudinal studies or studies comparing adolescent and adult samples with AN may help to gain insight into the different role body weight seems to play for body dissatisfaction and self-esteem.

A different pattern emerged in HC girls: Actual weight (BMI-SDS) was a positive predictor of body dissatisfaction and a negative predictor of self-esteem, which is in line with our hypotheses and previous research showing that adolescents with lower weight (i.e., those who meet societal thinness ideals) tend to experience lower body dissatisfaction and higher self-esteem [37, 50, 59]. Importantly, the addition of interpretation bias to the models significantly increased the model fit: Together, actual weight and interpretation bias explained 31% of variance in body dissatisfaction and 42% of variance in self-esteem. Negative interpretation bias was a positive predictor for body dissatisfaction and a negative predictor for self-esteem. This indicates that cognitive processes are related to body dissatisfaction and self-esteem also in adolescents without mental disorder, but less strongly than in adolescents with AN. In girls without mental disorder, body dissatisfaction and self-esteem seem to be linked to both their actual weight, i.e., the physical condition of their bodies, *and* cognitions about their bodies.

Importantly, the cross-sectional design of this study prevents conclusions about causality, temporal relationships, or changes related to weight loss or the course of AN, which is a major limitation of the present investigation. Consequently, it is not possible to disentangle whether the observed characteristics are core AN symptoms, their cognitive correlates, or mechanisms linking them, within the present study. For example, while cognitive biases may lead to body dissatisfaction and low self-esteem in adolescents with AN, it is equally plausible that body dissatisfaction and low self-esteem play a role in the development of (negative) cognitive biases. To date, it remains uncertain whether body-related negative cognitive biases contribute to the onset of AN or if they emerge as a result of the disorder. The same applies to body dissatisfaction and low self-esteem: All may represent predisposing factors, consequences, or maintaining mechanisms - or all of these. In adolescents without mental disorder, low weight may lead to reduced body dissatisfaction and improved self-esteem, while it is equally plausible that body dissatisfaction and low self-esteem prompt intentional dietary changes and weight loss. Furthermore, one could also assume non-linear relations between the different factors. For instance, weight loss may initially lead to an increase in self-esteem and body satisfaction (resulting in the associations found in the HC sample) but as weight loss progresses and is accompanied by eating disorder symptoms, cognitive biases may emerge or aggravate, consequently interfering with the perception of the body as lean and reinforcing the perception of the body as flawed. As a result, further weight loss may no longer lead to an increase in self-esteem or body satisfaction. It is also possible that the speed of weight loss has an influence on this relationship. To understand the role of these potential mechanisms in the development and maintenance of AN, future studies should use longitudinal designs (e.g., observe changes in weight, body (dis)satisfaction, and self-esteem over a longer period of time in adolescents with and without AN). In addition, experimental studies (e.g., modifying cognitive biases and assessing the influence of this modification on body (dis)satisfaction and self-esteem) are necessary to investigate causal relationships.

Further, to better understand the complex associations between body weight, self-esteem, and body dissatisfaction in adolescents with AN, future studies should consider potentially mediating factors. For instance, research in adults with AN suggests that weight loss in AN patients may provide them with a sense of achievement [52] which might act as an internal reinforcer for eating disorder behavior and as such might play a more critical role in maintaining AN than external reinforcers such as admiration from others [99, 100]. Individuals with AN may have initially pursued weight loss to improve body satisfaction or self-esteem, however, due to the internal reinforcement they experience (feelings of accomplishment), they may continue their efforts to lose weight, even after reaching a point where weight loss no longer provides benefits for body satisfaction or self-esteem.

It should be noted that although the original study included three groups – AN, HC, and a clinical control group – only the AN and HC groups were included in the present investigation. This decision was based on the substantial overlap in symptoms as well cognitive biases between AN and clinical control groups, which would have limited the interpretability of group-specific results. However, since low self-esteem, body dissatisfaction, as well as negative cognitive biases for body-related information are all transdiagnostic characteristics, the pattern of results found in adolescents with AN might not be unique to this group but also apply to adolescents with different mental disorders that overlap in some of these characteristics (see also results in the clinical control group in the supplement ).

In terms of limitations of the present study, it needs to be mentioned that only female adolescents were included in the present study, some of the questionnaire measures are validated mostly in female populations (e.g., the BSQ [83]), and interpretation bias was assessed using stimuli that targeted the typical thin body ideal found in most females with AN. Therefore, our results may not be generalizable to male adolescents with AN, given that body dissatisfaction is often linked to muscularity rather than thinness in males [101]. Furthermore, all subjects in the AN group were currently undergoing psychotherapy, which might have had an impact on the investigated characteristics (i.e., self-esteem, body dissatisfaction, cognitive biases). Moreover, the small sample size of our study might have resulted in the failure to detect smaller effects. Additionally, we did not examine potential differences in the predictive power of weight and body-related cognitions across different weight categories (e.g., overweight) or eating disorder diagnoses, which differ, e.g., regarding weight-loss behavior and drive for thinness. Future studies should therefore expand the sample size and differentiate between different weight categories and eating disorder diagnoses to gain a deeper understanding of the underlying mechanisms.

### Clinical implications

The present study’s findings carry potential implications for the treatment of AN. The observed association between biased cognitions and body dissatisfaction/self-esteem indicates that cognitive biases could be a target of therapeutic interventions. Cognitive Bias Modification (CBM) trainings [102, 103] are a promising tool to modify cognitive biases directly and could be administered in addition to cognitive restructuring in the context of Cognitive-Behavioral Therapy (CBT) that can be used to modify underlying maladaptive schemata. Addressing the dysfunctional cognitions related to one’s body may facilitate interrupting dysfunctional associations between low self-esteem, body dissatisfaction, and disordered eating behaviors.

In HC adolescents, both body weight and cognitions about the body predicted body (dis)satisfaction and self-esteem. This suggests that both could be considered in efforts to prevent AN and other eating disorders. Preventive programs (see [104] for a meta-analysis) could help adolescents to develop a healthier perspective on their body and challenge harmful societal messages by (i) encouraging adolescents to value themselves beyond their physical appearance, fostering self-esteem through various attributes like talents, skills, and personal qualities, rather than relying primarily on characteristics of the body; (ii) helping adolescents to appreciate and accept their bodies, fostering a more positive body image and reducing the likelihood of dissatisfaction in the first place; and (iii) providing tools for dealing with negative thoughts about their bodies that are reinforced by (social) media portrayals of beauty.

## Conclusion

The present study’s findings show how negative body-related interpretation biases might function as a psychological mechanism underlying body dissatisfaction and low self-esteem in adolescents with AN. These cognitive biases may partially account for the disconnection between the physical reality of the body and the evaluation of the body and the self in adolescents with AN, who, unlike adolescents without mental disorder, are often highly dissatisfied with their bodies despite low body weight. However, the cross-sectional design of our study prohibits conclusions about causality or temporal relationships so our results can only be considered a starting point for future longitudinal and experimental studies investigating the suggested mechanisms more comprehensively.

## Electronic supplementary material

Below is the link to the electronic supplementary material.

Supplementary Material 1

## Data Availability

The data are available from the corresponding author on reasonable request.

## Footnotes

1 The KOALA-study systematically examined cognitive biases in adolescents with AN compared to a clinical control group (adolescents with depression and/or anxiety disorders who did not meet criteria for any current or past eating disorder as assessed with the Kinder-DIPS), and a healthy control group (HC). Cognitive biases were assessed on three information processing levels (attention, interpretation, and memory) and for two types of information (biases for eating disorder-related and non-eating disorder-related information) within a single experimental paradigm. Both clinical groups (AN and clinical controls) displayed more pronounced negative interpretation and memory biases than the HC group across both types of information while not differing significantly from each other, neither in bias scores nor in symptom measures [75], underlining that cognitive biases as well as certain eating disorder symptoms are transdiagnostic phenomena. Biases for eating disorder-related and non-eating disorder-related information were highly correlated (*r* =.81 for interpretation biases and *r* =.52 for memory biases; [75]). Due to these large overlaps between the two clinical groups on the one hand and biases for the two types of information on the other hand, we decided a priori to include only the two distinguishable groups (AN and HC) and only biases for eating disorder-related information (being the more disorder-specific measure) in the present investigation. Nevertheless, we provide the results for all groups and biases in the supplement.

2 Two participants scored just below 85 but were included nonetheless, as this did not change the pattern of results.

3 All *R²* were reported as adjusted scores; >0.26 indicates a high goodness-of-fit according to Cohen [94].

## Abbreviations

AN: Anorexia nervosa
BMI: Body mass index
BMI-SDS: Body mass index standard deviation score
BSQ: Body shape questionnaire
CFT-20-R: Culture fair intelligence test revised
DSM-5: Diagnostic and statistical manual of mental disorders, fifth edition
EDI: Eating disorder inventory-2
HC: Healthy control
Kinder-DIPS: Diagnostisches interview bei psychischen Störungen im kindes- und jugendalter [diagnostic interview for mental disorders in childhood and adolescence
RSES: Rosenberg self-esteem scale
SST: Scrambled sentence task
