# Supplementary material for: Losing weight, gaining confidence? actual weight does not predict body (dis)satisfaction and self-esteem in adolescents with anorexia nervosa
Source: J Eat Disord. 2025 Jul 16;13:141. doi: 10.1186/s40337-025-01338-3 (PMC12265378; doi:10.1186/s40337-025-01338-3)
Supplement: Supplementary file 1 — Supplementary Material 1 [file 40337_2025_1338_MOESM1_ESM.pdf]

Supplement to:
Losing weight, gaining confidence? Actual weight does not predict body (dis)satisfaction and self-esteem in adolescents with anorexia nervosa

SUPPLEMENT

Supplement 1: Memory bias

In the KOALA study not only interpretation biases but also memory biases were assessed and more negative memory biases were found in adolescents with AN compared to adolescents without mental disorder (Lukas et al., 2024). As memory biases were highly correlated to and not independent from interpretation biases, they were not included in the main analyses of the present study. However, we repeated our analyses with memory biases instead of interpretation biases as a measure for cognitive biases.

The Scrambled Sentences Task (SST) was followed by a filler task, after which participants were asked to recall as many of the sentences they had previously formed das possible (incidental free recall task). Memory bias scores were calculated analogous to interpretation bias scores, as the proportion of negative sentences from all correctly remembered sentences. Hierarchical linear regressions with weight (BMI-SDS; first step) and body-related memory bias (second step) as predictors for self-esteem and body dissatisfaction (outcome variables) were conducted.

In the AN group, the hierarchical regression revealed BMI-SDS not predicting a significant amount of variance in body dissatisfaction ($F_{1,36}$ = 0.31, p = .580, R^2 = -.02), but when entering memory bias into the model, the explained variance increased significantly (ΔR^2 = .16, p = .014; $F_{1,35}$ = 3.56, p = .039, R^2 = .12). Results for self-esteem show the same pattern: BMI-SDS was no significant predictor for self-esteem ($F_{1,37}$ = 0.12, p = .735, R^2 = -.02), but when adding body-related memory bias to the model, a significant increase of the explained variance was found (ΔR^2 = .36, p <.001; $F_{1,36}$ = 10.13, p < .001, R^2 = .33) In the HC group, BMI-SDS alone significantly explained a significant amount of variance of body dissatisfaction ($F_{1,33}$ = 7.78, p = .009, R^2 = .17). Adding memory bias in the second step increased the proportion of explained variance significantly (ΔR^2 = .17, p = .006; $F_{1,32}$ = 9.12, p < .001, R^2 = .32). BMI-SDS also predicted self-esteem ($F_{1,32}$ = 5.35, p = .027, R^2 = .12), but when adding memory bias in the second step, the proportion of explained variance increased significantly (ΔR^2 = .26, p < .001; $F_{1,31}$ = 10.64, p < .001, R^2 = .37). See Supplementary Table 1 for detailed results on the predictors.

Supplement to:
Losing weight, gaining confidence? Actual weight does not predict body (dis)satisfaction and self-esteem in adolescents with anorexia nervosa

Supplementary Table 1

Results of the regression analysis in the both groups

	B	*SE for B*	*95 % CI for B*	*β*	*t*	*p*
AN						
Body dissatisfaction (BSQ)[a]						
STEP 1						
Weight (BMI-SDS)	-4.71	8.42	[-21.78, 12.36]	-.09	-0.56	.580
STEP 2						
Weight (BMI-SDS)	-1.86	7.89	[-17.88,14.16]	-.04	-0.24	.815
Memory bias	42.11	16.20	[9.23, 75.00]	.41	2.60	**.014**
Self-esteem (RSES)[b]						
STEP 1						
Weight (BMI-SDS)	.52	1.54	[-2.59, 3.63]	.06	0.34	.735
STEP 2						
Weight (BMI-SDS)	-0.24	1.26	[-2.79, 2.31]	-.03	-0.19	.850
Memory bias	-11.62	2.59	[-16.87, -6.36]	-.60	-4.48	**<.001**
HC						
Body dissatisfaction (BSQ)[c]						
STEP 1						
Weight (BMI-SDS)	15.91	5.70	[4.31, 27.51]	.44	2.79	**.009**
STEP 2						
Weight (BMI-SDS)	15.37	5.14	[4.90, 25.84]	.42	2.99	**.005**
Memory bias	44.43	15.11	[13.65, 75.21]	.42	2.94	**.006**
Belf-esteem (RSES)[d]						
STEP 1						
Weight (BMI-SDS)	-2.00	.86	[-3.75, -0.24]	-.38	-2.31	**.027**
STEP 2						
Weight (BMI-SDS)	-1.94	.73	[-3.43, -0.45]	-.37	-2.66	**.012**
Memory bias	-7.84	2.11	[-12.14, -3.53]	-.51	-3.71	**<.001**

Note. AN = anorexia nervosa; HC = Healthy control; BMI-SDS = body mass index standard deviation score; BSQ = Body shape questionnaire; RSES = Rosenberg self-esteem scale.
[a] available from n = 38 participants.
[b] available from n = 39 participants.
[c] available from n = 35 participants.
[d] available from n = 34 participants.

Supplement to:
Losing weight, gaining confidence? Actual weight does not predict body (dis)satisfaction and self-esteem in adolescents with anorexia nervosa

Supplement 2: Results of the clinical control group

The regression revealed that BMI-SDS alone did not account for a significant amount of variance in body dissatisfaction ($F_{1,29}$ = 1.46, p = .237, R^2 = .015), but when entering interpretation bias, the model was significant ($F_{2,28}$ = 8.66, p < .001, R^2 = .338) with interpretation bias being a significant positive predictor for body dissatisfaction in this group. The same pattern was found for self-esteem: BMI-SDS alone did not account for a significant amount of variance in self-esteem ($F_{1,29}$ = 1.39, p = .248, R^2 = .013). When interpretation bias was added, the model explained a significant proportion of variance ($F_{2,28}$ = 5.92, p = .007, R^2 = .25) and interpretation bias was a significant negative predictor for self-esteem. See Supplementary Table 2 for detailed results on the predictors.

Supplementary Table 2

Results of the regression analyses with eating disorder-related interpretation in the CC Group

	B	*SE for B*	*95 % CI for B*	*β*	*t*	*p*
CC (clinical control group)						
<u>Body dissatisfaction (BSQ)</u>						
STEP 1						
Weight (BMI-SDS)	9.15	7.57	[-6.34, 24.63]	.22	1.21	.237
STEP 2						
Weight (BMI-SDS)	3.46	6.38	[-9.61,16.52]	.08	0.54	.592
Interpretation bias	72.08	18.52	[34.14, 110.01]	.59	3.89	**<.001**
<u>Self-esteem (RSES)</u>						
STEP 1						
Weight (BMI-SDS)	1.47	1.25	[-1.08, 4.02]	.21	1.18	.248
STEP 2						
Weight (BMI-SDS)	2.28	1.12	[-0.01, 4.57]	.33	2.04	.051
Interpretation bias	-10.28	3.25	[-16.93, -3.62]	-.52	-3.16	**.004**

Note. BMI-SDS = body mass index standard deviation score; BSQ = Body shape questionnaire; RSES = Rosenberg self-esteem scale

Supplement to:
Losing weight, gaining confidence? Actual weight does not predict body (dis)satisfaction and self-esteem in adolescents with anorexia nervosa

Supplement 3: Results for non-eating disorder-related biases

Regression analyses with non-eating disorder-related interpretation bias as predictor yielded the following results. In the AN group, the regression revealed that BMI-SDS alone did not account for a significant amount of variance in body dissatisfaction ($F < 1$), but when entering non-eating disorder-related interpretation bias, the model was significant ($F_{1,36}$ = 6.32, p = .004, R^2 = .22) with interpretation bias being a significant positive predictor for body dissatisfaction. We found the same pattern for self-esteem: BMI-SDS alone did not account for a significant amount of variance in self-esteem ($F < 1$). When non-eating disorder-related interpretation bias was added, the model explained a significant proportion of variance ($F_{1,37}$ = 27.10, p < .001, R^2 = .59) and interpretation bias was a significant negative predictor of self-esteem. In the HC group, BMI-SDS alone accounted for a significant amount of variance in body dissatisfaction ($F_{1,35}$ = 9.01, p = .005, R^2 = .18). When non-eating disorder-related interpretation bias was added into the model, the explained variance did not increase significantly (ΔR^2 = .04, p = .192; $F_{1,34}$ = 5.49, p = .009, R^2 = .24). BMI-SDS did also explain a significant amount of variance in self-esteem ($F_{1,34}$ = 4.74, p = .037, R^2 = .10). Adding non-eating disorder-related interpretation bias increased the proportion of explained variance significantly (ΔR^2 = .29, p < .001; $F_{1,33}$ = 11.33, p < .001, R^2 = .41). Both BMI-SDS and interpretation bias were significant negative predictors of self-esteem in this final model. In the clinical control group, the regression revealed that BMI-SDS did not account for a significant amount of variance in body dissatisfaction ($F_{1,28}$ = 1.46, p = .237, R^2 = .02). When entering non-eating disorder-related interpretation bias, the model remained non-significant (ΔR^2 = .10, p = .085; $F_{1,28}$ = 2.38, p = .111, R^2 = .08). A different pattern emerged for self-esteem: BMI-SDS alone did not account for a significant amount of variance in self-esteem ($F_{1,28}$ = 1.39, p = .248, R^2 = .01) but when non-eating disorder-related interpretation bias was added, the model explained a significant proportion of variance (ΔR^2 = .26, p = .003; $F_{1,28}$ = 6.08, p = .006, R^2 = .25) and interpretation bias was a significant negative predictor for self-esteem. See Supplementary Table 3 for detailed results.

Supplement to:
Losing weight, gaining confidence? Actual weight does not predict body (dis)satisfaction and self-esteem in adolescents with anorexia nervosa

Supplementary Table 3

Results of the regression analyses with non-eating disorder-related interpretation in all groups.

	B	*SE for B*	*95 % CI for B*	*β*	*t*	*p*
AN						
Body dissatisfaction (BSQ)						
STEP 1						
Weight (BMI-SDS)	-6.17	8.45	[-23.30, 10.95]	-.12	-0.73	.470
STEP 2						
Weight (BMI-SDS)	3.74	7.46	[-18.87,11.38]	-.07	-0.50	.619
Interpretation bias	72.40	20.94	[29.93, 114.86]	.50	3.46	**.001**
Self-esteem (RSES)						
STEP 1						
Weight (BMI-SDS)	.58	1.50	[7.23, 20.45]	.06	0.382	.704
STEP 2						
Weight (BMI-SDS)	0.01	0.98	[-1.99, 1.99]	.00	0.10	.992
Interpretation bias	-19.95	2.72	[-25.46, -14.44]	-.78	-7.24	**<.001**
HC						
Body dissatisfaction (BSQ)						
STEP 1						
Weight (BMI-SDS)	14.04	4.68	[4.54, 23.54]	.45	3.00	**.005**
STEP 2						
Weight (BMI-SDS)	15.51	4.76	[5.48, 23.01]	.50	3.26	**.003**
Interpretation bias	27.07	20.35	[9.82, 71.37]	.20	1.33	.192
Self-esteem (RSES)						
STEP 1						
Weight (BMI-SDS)	-1.60	.736	[25.38, 27.89]	-.35	-2.18	**.037**
STEP 2						
Weight (BMI-SDS)	-2.17	.630	[-3.46, -0.89]	-.47	-3.45	**.002**
Interpretation bias	-10.62	2.67	[-16.05, -5.20]	-.55	-3.98	**<.001**
CC (clinical control group)						
Body dissatisfaction (BSQ)						
STEP 1						
Weight (BMI-SDS)	9.15	7.57	[-6.34, 24.63]	.22	1.21	.237
STEP 2						
Weight (BMI-SDS)	11.90	7.46	[-3.39, 27.19]	.29	1.60	.122
Interpretation bias	49.28	27.63	[-7.31, 105.87]	.32	1.78	.085
Self-esteem (RSES)						
STEP 1						
Weight (BMI-SDS)	1.47	1.25	[25.38, 27.89]	.21	1.18	.248
STEP 2						
Weight (BMI-SDS)	0.73	1.11	[-2.85, -0.46]	.11	0.66	.514
Interpretation bias	-13.18	4.10	[-13.34, -5.04]	-.52	-3.21	**.003**

AN = anorexia nervosa; HC = healthy control; CC = clinical control group; BMI-SDS = body mass index standard deviation score; BSQ = Body shape questionnaire; RSES = Rosenberg self-esteem scale.
